# From Low-Resource Innovation to High-Resource Learning: Head-Mounted Cameras as a Tool to Strengthen Surgical and Burn Care Training

**DOI:** 10.3390/ebj7020020

**Published:** 2026-04-01

**Authors:** Einar Logi Snorrason, Fredrik Huss, Ali Modarressi, Morten Kildal

**Affiliations:** 1 Burn Center, Department of Plastic and Maxillofacial Surgery, Uppsala University Hospital, 751 85 Uppsala, Sweden; fredrik.huss@uu.se (F.H.); morten.kildal@uu.se (M.K.); 2 Department of Surgical Sciences, Uppsala University, 751 05 Uppsala, Sweden; 3 2nd Chance Association, 1207 Geneva, Switzerland

**Keywords:** burn(s), burn care, education, telemedicine, wearable technology, head-mounted camera, patient safety, surgical training

## Abstract

While the global surgeon deficit continues to demand urgent action, traditional “over-the-shoulder” teaching is increasingly constrained by infection-control demands and crowded operating rooms. Over the past four years, we integrated head-mounted smart cameras into reconstructive-surgery workshops across East Africa. Utilizing voice-controlled, stabilized video technology, we provided trainees with a high-definition, wearer’s-perspective view that enhanced visualization without compromising the sterile field. Following remarkably high acceptance in Africa, we have initiated a pilot study at the National Burn Centre in Sweden to apply these lessons to a high-income setting. Our findings suggest that this technology improves surgical education while supporting infection-control stewardship through reduced overcrowding. This experience illustrates a reverse innovation, where tools refined under the logistical constraints of African operating theatres offer scalable solutions for universal challenges in surgical training and patient safety.

## 1. Introduction

The global deficit in trained surgeons and anesthetists remains a critical barrier to safe and timely surgery. Despite increasing awareness since the Lancet Commission on Global Surgery in 2015, nine of ten people in low- and middle-income countries (LMICs) still lack access to essential surgical care [1]. A 2025 update in The Lancet highlighted this crisis, warning that global progress is off track, with an increase in the unmet need for surgery [2]. Efforts to expand surgical capacity are largely dependent on efficient training, as traditional “over-the-shoulder” teaching inside crowded operating theatres is constrained by infection-control demands, scarce faculty time, and limited viewing opportunities. In this context, video-based learning has emerged as a vital adjunct, having been shown to enhance surgical education through knowledge retention, and to improve technical skills [3,4,5,6]. This transition has also been evident in Swedish medical universities, with a recent nationwide survey confirming that students have extensively embraced digital learning resources and video-based tools as core components of their education [7]. Modern telemedicine and wearable video technologies now offer a way to rethink surgical training—enhancing safety through improved infection stewardship and reduced overcrowding, while remaining interactive and scalable [8,9]. Notably, while global health initiatives often focus on transferring technology from high- to low-resource settings, there is an increasing recognition of reverse innovation [10].

## 2. Current Developments

**Figure 1:**
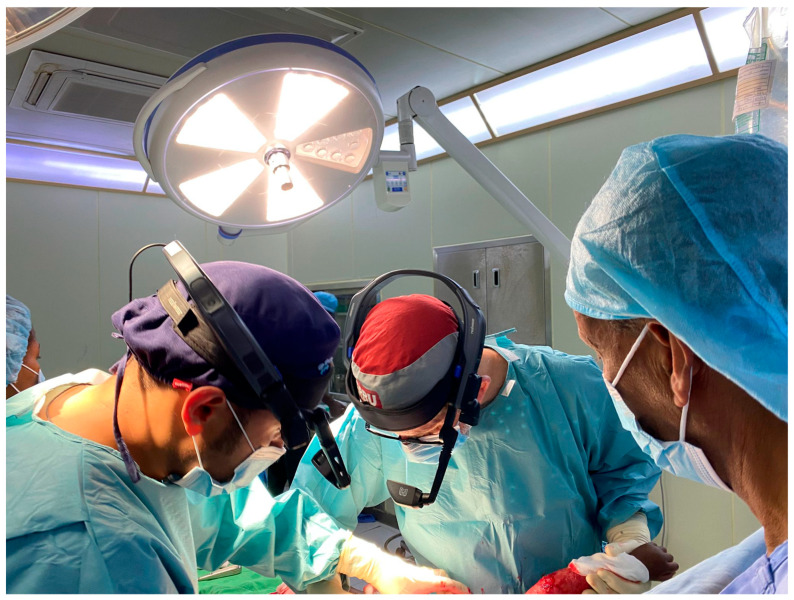
Head-mounted camera system in an intraoperative setting. Surgeons are equipped with wearable camera units positioned to capture the surgical field from a first-person perspective.

**Figure 2:**
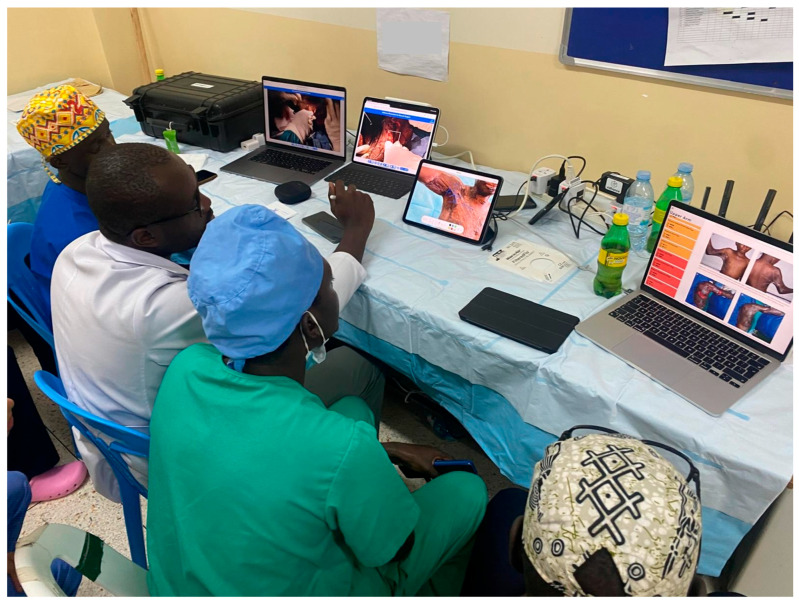
Live surgical training in an adjacent room. Trainees utilize tablets to observe the procedure remotely, allowing for interactive learning and simultaneous access to educational resources.

Over the past four years, our team has employed head-mounted smart cameras in reconstructive-surgery workshops across Uganda, Kenya and Ethiopia in collaboration with 2nd Chance Association, a Swiss non-profit organization dedicated to developing reconstructive surgery teams in low-resource settings [11]. The system used (RealWear^®^ hardware (Vancouver, WA, USA) with Vidhance^®^ for Surgery 1.0 software (Uppsala, Sweden)) securely transmits high-definition, stabilized, real-time video and two-way audio from the wearer’s perspective (Figure 1). Trainees observe on local tablets in an adjacent room, allowing live discussion without compromising the sterile area or overcrowding the procedure room (Figure 2).

Preliminary feedback collected from surgeons and anesthesiologists in eight African countries revealed a remarkably high level of acceptance. The most frequently mentioned advantage was enhancement of the overall learning experience. This was reflected in high levels of engagement, with more than half of the participants feeling very actively involved and one-third feeling much involved while observing from the viewing room. Only a tenth remained neutral. Participants also reported improved visualization, better focus during teaching discussions, and enhanced patient safety through reduced overcrowding. Trainers valued the ability to teach larger groups simultaneously, while trainees appreciated a structured and comfortable learning environment. These preliminary findings indicate that telemedicine—when developed in close dialogue with end-users—can strengthen both education and safety in low-resource surgical settings.

## 3. Future Directions

Our experiences described above also highlight the paradox that in highly digitalized university hospitals, such as those found in Sweden, similar head-mounted systems have not been adopted. The reason seems less technical than contextual: many available cameras were never developed from the actual educational and ergonomic needs of surgical teams. For this reason, we have initiated a pilot study at the National Burn Centre, Uppsala University Hospital, to implement and evaluate the same LMIC-tested system within a high-income environment. This initiative represents a “win–win” collaboration, bringing back lessons learned under constrained conditions to address challenges that are surprisingly universal.

Burn care is uniquely dependent on visual analysis. Developing a trained clinical eye demands repeated exposure to diverse wounds, yet opportunities for direct observation during dressing changes or surgery are often limited. Infection-control protocols restrict the number of personnel in procedure rooms due to e.g., contamination risks. The intricate synchronization of morning workloads, fasting requirements, and unpredictable patient deterioration further reduce teaching time. Increasing the number of observers in the procedure room may also paradoxically compromise workflow. Thus, a persistent question remains: How can we effectively train competent staff while maintaining strict infection control and efficiency?

Our hypothesis is that wearable, head-mounted cameras can relieve this tension. In our pilot, a surgeon or nurse wears the device during operations, dressing changes or hydrotherapy sessions, streaming stabilized video and two-way audio to team members gathered in the adjacent nurses’ station which is only accessible by relevant personnel. The system is voice-controlled, hands-free, and requires only a brief setup. Beyond real-time observation, this technology offers future potential for archiving high-definition footage for the purpose of asynchronous training and to enable remote supervision of senior trainees, allowing consultants to provide guidance without physical presence.

## 4. Discussion

Our initial experiences have provided key insights into the system’s integration. Preliminary feedback is encouraging, highlighting the system’s clear, stable imagery and seamless communication. The device does not interfere with Personal Protective Equipment (PPE), it feels lightweight, and it offers accurate color rendering even under strong operating-room lights. Communication between wearer and observers is immediate, and the local-network configuration avoids reliance on hospital Wi-Fi whilst ensuring data security. Observers can request alternative angles on what is seen, or clarification of what is happening, without entering the room or sterile field, whilst the wearer can describe wound characteristics and reasoning in real time. We anticipate potential benefits in both workflow efficiency, by reducing unnecessary entries and PPE changes, and in infection control stewardship by minimizing door openings for supply retrieval and consultations that are known risks [12]. The pilot study is designed strictly for live, interactive training; video and audio are therefore streamed in real-time, without recording. However, baseline characteristics will be collected for statistical analysis, requiring informed consent from both patients and participating personnel. Although quantitative data are forthcoming, the qualitative response has been strongly positive, suggesting improvements in teamwork, situational awareness, and educational reach.

Perhaps most importantly, this experience illustrates reverse innovation in global health. Technologies refined under the constraints of African operating theatres, where reliability, simplicity, and cost-efficiency are mandatory, may also help solve educational and logistical problems in advanced hospitals. Such bidirectional learning reinforces sustainable partnerships and mutual respect, where each setting contributes to the other’s progress.

Challenges remain. These include avoiding cognitive overload for the wearer, potentially integrating video with patient record systems, and preserving the essential hands-on mentorship that defines surgical learning. Potential solutions to these challenges include mitigating cognitive overload through voice-commanded system customization, which allows surgeons to regulate incoming audio volume from the viewing room, control when to engage in verbal communication, and determine exactly what visual content is shared. These aspects will be explored in our forthcoming evaluation, using both qualitative and quantitative methods. Nevertheless, early experience suggests that head-mounted cameras can become a valuable adjunct to bedside teaching by enhancing communication, maintaining infection control stewardship, and expanding access to high-quality visual learning without compromising professionalism or patient safety.

## 5. Conclusions

As global surgery advances toward greater equity, tools that allow clinicians to see through each other’s eyes may prove indispensable. The convergence of telemedicine, burn-care education, and reverse innovation offers a realistic, scalable way forward, born in low-resource settings and now transitioning to enrich high-resource healthcare.

## Data Availability

No new data were created or analyzed in this study.
